# Molecular detection of rabies virus strain with N-gene that clustered with China lineage 2 co-circulating with Africa lineages in Monrovia, Liberia: first reported case in Africa

**DOI:** 10.1017/S0950268818003333

**Published:** 2019-02-19

**Authors:** A. O. Olarinmoye, V. Kamara, N. D. Jomah, B. O. Olugasa, O. O. Ishola, A. Kamara, P. D. Luka

**Affiliations:** 1 Eng. Abdullah Bugshan Research Chair for Dental and Oral Rehabilitation (D.O.R), College of Dentistry, King Saud University, Riyadh, Kingdom of Saudi Arabia; 2 Center for Control and Prevention of Zoonoses, Faculty of Veterinary Medicine, University of Ibadan, Ibadan, Nigeria; 3 Department of Veterinary Public Health and Preventive Medicine, University of Ibadan, Ibadan, Nigeria; 4 Ministry of Agriculture, Leon Quest Ledlum Central Veterinary Diagnostic Laboratory, Fendel, Monrovia, Liberia; 5 Central Agricultural Research Institute (C.A.R.I.), Suakoko, Bong County, Liberia; 6 Ministry of Agriculture Sub-office, Buchanan, Grand Bassa County, Liberia; 7 National Veterinary Research Institute (N.V.R.I.), Vom, Plateau State, Nigeria

**Keywords:** Liberia, Monrovia, *Rabies* (canine), RT-PCR, sequencing analysis

## Abstract

Despite a long history of dog-transmitted human rabies outbreaks in Liberia, West Africa, no reports exist of molecular characterisation of the causative lyssaviruses. This study investigated *Rabies lyssavirus* (RABV) strains isolated at the dog–human interface in Monrovia, Liberia 2016 and 2017, by reverse transcription polymerase chain reaction, using primers specific for the nucleoprotein (N) gene. Out of 20 specimens (19 dog brain samples and one human saliva) tested as suspected rabies cases, three (15%) were positive. Purified amplicons from all three positive specimens were sequenced in both forward and reverse directions. Phylogenetic analysis was conducted in MEGA7 and PhyML3 to determine their relationship with RABV sequences accessioned in NCBI GenBank. The first of three RABV strains detected clustered with China lineage 2 RABVs of dogs (99% homology to KU963489 and DQ666322). The second strain segregated with Africa lineage 2 RABVs also of dog origin, and the third strain segregated with Africa lineage 3 RABVs of Southern Africa viverrids. Our results show a transcontinental strain of rabies virus co-circulating with Africa lineages in post-conflict Liberia. This finding should stimulate more effective sub-regional planning and execution of one-health actions, towards stepwise surveillance and elimination of rabies in West Africa by 2030.

## Introduction

The goal of improving human–animal disease surveillance in West Africa remains an active concept note towards stepwise elimination of rabies in Africa by 2030 [1]. Although it has been more than seven decades since the first medical report of clinical diagnosis of rabies in dog bite victims (DBVs) in Liberia [2], the disease remains a neglected public health challenge associated with acute, progressive and highly fatal viral encephalomyelitis of warm-blooded animals (especially dogs) and humans [3, 4]. The extremely high rabies case fatality rate of nearly 100% makes it the deadliest of all known infections that are transmissible between animals and human beings. Yet, in post-conflict Liberia where dog-transmitted human rabies (DTHR) is enzootic, active surveillance for cases has been relatively neglected [3, 4], and the country is regarded as one of the remaining dark corners of rabies in Africa [5]. Periodic evaluation of the rabies status of many developing world countries such as Liberia is hampered by misdiagnosis, under-reporting, poor surveillance and unreliable data gathering at the local level [5]. However, with the emergence of Rabies in West Africa (RIWA) forum [6], there has been an increase in scientific studies and reports on rabies at the human–animal interface at various locations in Liberia, including Lofa County in the north, Bong County in the north-central and Grand Bassa County in the west-central region of the country [3], and also in Montserrado County, home to Monrovia the political capital of the country in the northwest region [4].

The high incidence of DTHR cases in Liberia has been attributed to poor vaccination coverage of owned dogs, the large population of stray dogs in urban areas, weak enforcement of dog control laws and difficulties in the cold-chain maintenance of vaccines in the post-conflict era [4]. Other factors include poor health care-seeking behaviour of DBVs, the high cost of rabies post-exposure prophylaxis, and a tendency of some DBVs to seek indigenous rather than orthodox healthcare [4]. While it is known that rabies is caused by all the 16 virus species currently listed under the order Mononegavirales, family Rhabdoviridae and genus Lyssavirus (bullet-shaped, single-stranded, negative-sense RNA viruses), [7] little has been done to characterise those that are in circulation in Liberia. Elsewhere, lyssaviruses that have been characterised include Aravan lyssavirus; Khujand lyssavirus; Irkut lyssavirus; Rabies lyssavirus (RABV); Duvenhage lyssavirus; European bat lyssavirus type 1; European bat lyssavirus type 2; Bokeloh bat lyssavirus; Australian bat lyssavirus; Lagos bat lyssavirus (LBV); Mokola lyssavirus (MOKV); Shimoni bat lyssavirus; West Caucasian bat lyssavirus; Ikoma lyssavirus (IKOV); Gannoruwa bat lyssavirus; and Lleida bat lyssavirus [7]. Of these lyssaviruses, the prototype and most commonly incriminated species in rabies epizootics worldwide is RABV [8]. An estimated 60 000 human rabies deaths occur each year, mostly among DBVs who are below the age of 15 years and resident in developing countries of Africa and Asia where cases are grossly under-reported [9].

To our best knowledge, the species, strains and phylogeny of lyssaviruses responsible for human and animal rabies in Liberia have not been previously identified or reported in medical literature. This was evident in the complete absence of accessioned rabies virus genes from Liberia in the NCBI or any other gene bank, before the three deposited from this study. Since a majority of the reported human rabies cases in Liberia are traceable to the bites of suspected rabid dogs [2–4], we hypothesised that dog-adapted RABV variants are responsible for enzootic rabies in the country. In this study, we aimed to characterise the causative strains and phylogeny of rabies viruses at the dog–human interface in Liberia for the first time.

## Methods

### Ethical approval

The ethical approval (EC/LIBR/014/039) entitled ‘Ethical approval for spatio-temporal epidemiology of suspected human cases of rabies, habitat suitability for rabies virus circulation, and molecular investigations, Liberia’ was granted by the Liberian Biomedical Research Institute (LIBR).

### Study locations

**Fig. 1.**
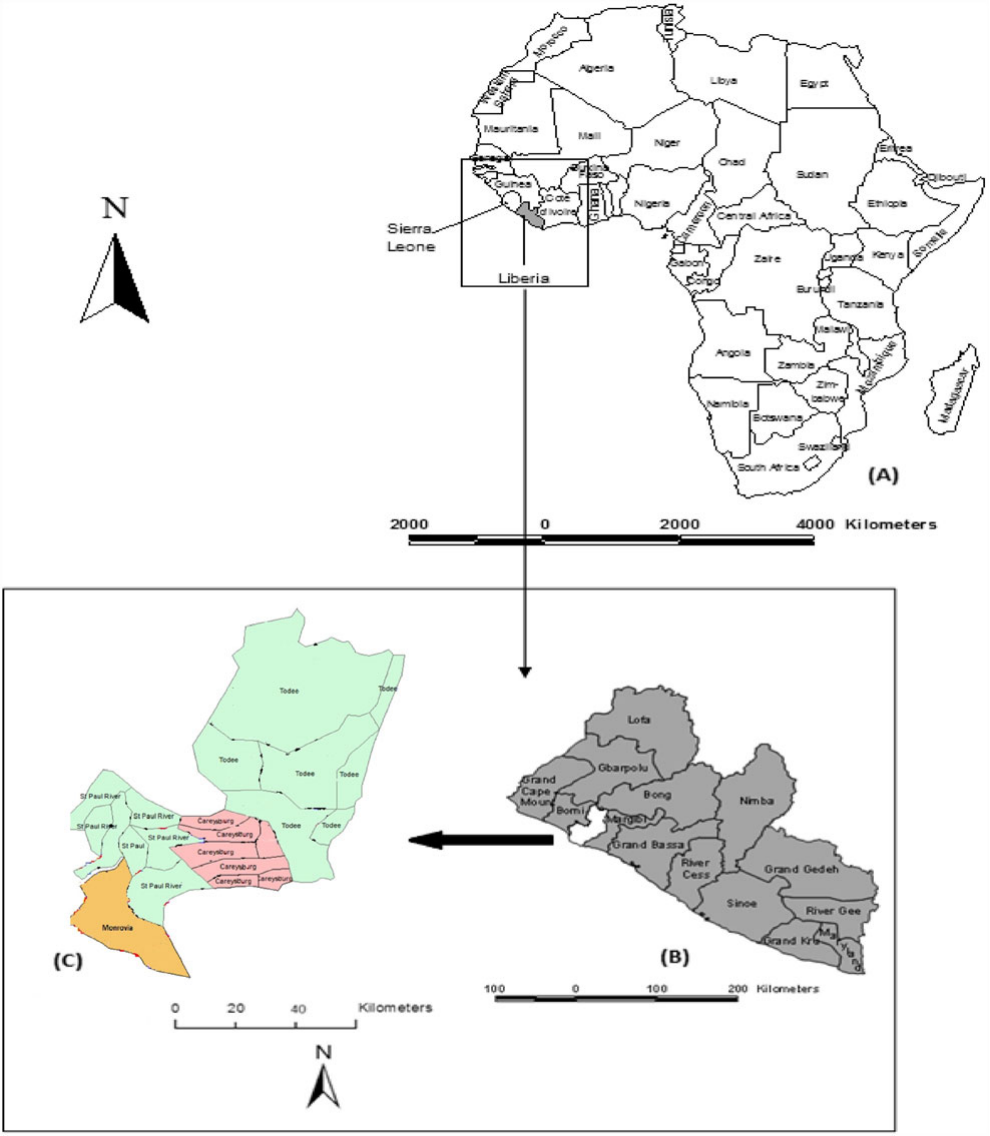
Map of Liberia in the West coast of Africa (a), Montserrado in the West coast of the country (b), and the four districts of Montserrado including Monrovia, the national capital (c).

The study was primarily conducted in Monrovia city (also known as Greater Monrovia), in Liberia (Fig. 1). Monrovia (population: 970 824) is located on latitude 6°18′48″N and longitude 10°48′5″W in Montserrado, the oldest of the 15 Counties of the government of the Republic of Liberia. Monrovia is the administrative and financial capital of Liberia, and also the most densely populated location in the entire country [10]. The climate of the area is tropical with a distinct wet season that lasts from May to November and a dry season that lasts from December to April, each year. A Central Veterinary Laboratory and Clinic (CVLC) is located in Fendel, a small town, East of Monrovia within Montserrado County.

The CVLC kept an archive of specimens that comprised brain tissues (mainly brain stem and hippocampus) of suspected rabid dogs (*n* = 110), killed during outbreaks of canine rabies within and around two Monrovia communities – Paynesville, including the Red Light neighbourhood, and Duala – from June to October 2016 and from September to October 2017.

### Passive surveillance of human–animal rabies in Liberia

The chronic neglect of rabies research in Liberia [11] received decisive attention by January 2012, when the University of Ibadan, Nigeria (UI) sponsored a 3-year project to improve postgraduate programmes for surveillance of human–animal diseases in West Africa. The programme enrolled career persons from the Ministries of Health, Agriculture and Universities in Liberia, Nigeria and Sierra Leone, into a one-health strategic surveillance of rabies programme, coordinated by the Centre for Control and Prevention of Zoonoses (CCPZ), University of Ibadan. The purpose was to address the paucity of verifiable rabies statistics in these West African countries, by gathering pertinent spatio-temporal and molecular data on human and animal cases of the disease. In furtherance of these activities, the RIWA forum was inaugurated at the University of Ibadan, Nigeria, December 2012 [6].

### Active surveillance for Lyssavirus species in Liberia

Active surveillance for Lyssavirus species in Liberia commenced in March 2014. At the time, there were no trained personnel and laboratory facilities for rabies diagnosis in Liberia. There were also no cold storage facilities available for the preservation of rabies suspect tissues. Against this backdrop, we decided to use Whatman^®^ FTA^®^ (Flinders Technology Associates) cards to collect and preserve rabies diagnostic samples including saliva, oro-pharyngeal secretions and brain tissues, for analysis at a later date. FTA cards have a filter paper component that is laced with proprietary chemicals that lyse cells and stabilise nucleic acids on contact, ensuring safe and long-term storage even at room temperature.

Serial saliva samples were obtained from a 10-year-old female DBV who was admitted to the Referral Clinic for Rabies Exposure in Monrovia, 2 months post-exposure with signs of clinical rabies. In April of 2014, the collection of rabies diagnostic samples was suspended in Liberia due to Ebola Virus Disease epidemic in the country. By the time sampling resumed in June 2016, the Liberian Government had set in motion a plan to upgrade the CVLC in Fendel, Monrovia. A solar-powered deep freezer facility and an archive of frozen dog brain tissue samples were soon established.

From the archive, a total of 19 dog brain samples were randomly selected for molecular tests to detect Lyssavirus infections. The samples were preserved in Ethanol 99% concentration, making them harmless and non-infections materials during shipment to laboratories in Nigeria where investigations aimed at detecting and determining the species, strain and phylogeny of the causative *Lyssavirus* were conducted at the Molecular Biology Laboratories of the Center for Control and Prevention of Zoonoses (CCPZ), University of Ibadan, Nigeria, and the National Veterinary Research Institute (NVRI), Vom, Plateau State, Nigeria. Sequencing of amplicons were conducted at the Biosciences Laboratory [11], International Institute of Tropical Agriculture (IITA), Ibadan, Nigeria. A summary presentation of the test specimens collected at the dog–human interface in Liberia during the course of field survey is presented in Table 1.

**Table 1.** Rabies diagnostic samples collected in Monrovia, Liberia for molecular and phylogenetic analysis

Serial No.	Lab. ID	Date of collection	Species	Source location	Types of sample		
					Brain tissues	Other	RT-PCR
1	CCPZUI/HS/35	22 March 2014	*Homo sapiens*	Paynesville, Monrovia, Liberia	No	Drool saliva^a^	Negative
2	DBS/004	20 June 2016	*Canis familiaris*	Red light, Paynesville, Monrovia, Liberia	Yes	No	Negative
3	DBS/01	15 October 2016	*Canis familiaris*	Duala, Monrovia North, Liberia	Yes	No	Positive; GenBank Accession No. MF765758
4	DBS/020	04 September 2017	*Canis familiaris*	Red light, Paynesville, Monrovia, Liberia	Yes	No	Negative
5	DBS/030	13 September 2017	*Canis familiaris*	Red light, Paynesville, Monrovia, Liberia	Yes	No	Negative
6	DBS/034	14 September 2017	*Canis familiaris*	Red light, Paynesville, Monrovia, Liberia	Yes	No	Negative
7	DBS/039	15 September 2017	*Canis familiaris*	Red light, Paynesville, Monrovia, Liberia	Yes	No	Negative
8	DBS/044	16 September 2017	*Canis familiaris*	Duala, Monrovia North, Liberia	Yes	No	Negative
9	DBS/052	21 September 2017	*Canis familiaris*	Red light, Paynesville, Monrovia, Liberia	Yes	No	Negative
10	DBS/067	27 September 2017	*Canis familiaris*	Duala (Monrovia North), Liberia	Yes	No	Negative
11	DBS/069	29 September 2017	*Canis familiaris*	Red light, Paynesville, Monrovia, Liberia	Yes	No	Negative
12	DBS/070	29 September 2017	*Canis familiaris*	Red light, Paynesville, Monrovia, Liberia	Yes	No	Negative
13	DBS/071	29 September 2017	*Canis familiaris*	Red light, Paynesville, Monrovia, Liberia	Yes	No	Negative
14	DBS/072	29 September 2017	*Canis familiaris*	Paynesville, Monrovia	Yes	No	Negative
15	DBS/073	02 October 2017	*Canis familiaris*	Paynesville, Monrovia	Yes	No	Negative
16	DBS/074	02 October 2017	*Canis familiaris*	Red light, Paynesville, Monrovia, Liberia	Yes	No	Negative
17	DBT/075	02 October 2017	*Canis familiaris*	Paynesville, Monrovia	Yes	No	Positive; GenBank Accession No. MH507336
18	DBS/076	02 October 2017	*Canis familiaris*	Paynesville, Monrovia	Yes	No	Positive; GenBank Accession No. MH507337
19	DBS/077	03 October 2017	*Canis familiaris*	Red light, Paynesville, Liberia	Yes	No	Negative
20	DBS/078	03 October 2017	*Canis familiaris*	Paynesville, Monrovia	Yes	No	Negative

a Three serial samples collected from the patient over a period of 6 h.

### Laboratory investigations

1. RNA extraction

One gram of brain tissue was homogenised and 9 ml of PBS was added and centrifuged in a refrigerated centrifuge at 10 000 rpm for 5 min to make 10% tissue suspension. Total RNA was extracted using QIAamp^®^ Viral RNA Mini Kit (QIAGEN, Hilden, Germany), according to the manufacturer's guidelines. The quality (260/280 nm) and concentration (ng/μl) of the extracted RNA was measured using the Eppendorf Biophotometer Plus^®^ UV-Visible Spectrophotometer (Eppendorf, Hamburg, Germany). The sample was immediately stored at −20 °C until further analysis.

1. Detection of the nucleoprotein (N) gene of lyssaviruses by reverse transcription polymerase chain reaction (RT-PCR)

Detection of the N gene of lyssaviruses by RT-PCR was performed using the GeneAmp^®^ Gold RNA PCR Core Kit (Applied Biosystems, Foster City, CA, USA), according to the manufacturer's guidelines. Three oligonucleotide primers namely JW12 (+), Lys001 (+) and 550B (+) were selected for use in this study, based on their annealing positions on Pasteur Virus (GenBank Accession Number M13215) (Table 2), as previously described [12, 13]. The primers were synthesised at Inqaba Biotechnical Industries (Pretoria, South Africa). The stages of the RT-PCR assay were as follows:

- Reverse transcription (RT)

**Table 2.** Primers used for RT-PCR assay for amplification of the *N* gene of lyssaviruses

Primer (sense)	Detection	Position according to Pasteur virus nucleoprotein (N) gene^a^	Nucleotide sequence 5′–3′	Purpose	References
001Lys (+)	RABV	1–15	ACGCTTAACGAMAAA	cDNA synthesis, PCR and sequencing	[12]
JW12 (+)	Pan-Lyssavirus	55–73	ATGTAACACCTCTACAATG	cDNA synthesis	[13]
550B (−)	RABV, LBV	647–666	GTRCTCCARTTAGCRCACAT	PCR and sequencing	[12]

a Pasteur virus (NCBI GenBank Accession Number M13215).

Complementary DNA (cDNA) was produced by RT reaction using the following protocol: 1.0 µl of forward primer JW12 (20 µm) was added to 1.0 µl of forward primer 001lys (20 µm), 7.0 µl of nuclease-free water (NFW) and 2 µl of total RNA. The mixture was incubated at 65 °C for 2 min and cooled on ice for 5 min, to heat denature and anneal it with the primers. This was followed by RT at 37 °C for 60 min, 85 °C for 5 min, 37 °C for 10 min, 72 °C for 5 min and cooling at 4 °C, in a final volume of 20 µl containing 2 µl. A 1× reverse transcriptase buffer, 4.0 µl of deoxynucleoside triphosphate (dNTP) mixture (10 mm), 0.5 µl MultiScribe™ Reverse Transcriptase (15 U/μl) and 0.5 µl of Ribonuclease inhibitor (20 U/μl).

1. PCR assay

Primary and secondary amplification of the cDNA (template) was performed using the primers 001lys and 550B, as previously described [12]. Briefly, a lyophilised anti-rabies vaccine (low egg passage Flurry Strain) from NVRI served as the positive control in the PCR. A non-template blank served as the negative control. Primary amplification of 5.0 µl of the cDNA template was performed in a final volume of 50 µl containing 4.0 µl of 001lys (20 µm), 4.0 µl of 550B (20 µm), 24.75 µl of NFW, 5.0 µl of 10× buffer, 3.0 µl of MgCl_2_ (25 mm), 4.0 µl of dNTP mixture (10 mm) and 0.25 µl of AmpliTaq polymerase (5 U/μl).

For the secondary amplification reaction mixture (50.0 µl), 5.0 µl of primary PCR product (1:50 dilution) was used as a template and the conditions repeated as for the primary amplification. Both amplification reactions were performed on a GeneAmp^®^ PCR System 9700 (Applied Biosystems). After denaturation at 95 °C for 1 min, reactions were cycled 40 times at 94 °C for 30 s, 37 °C for 30 s and 72 °C for 90 s, with final extension at 72 °C for 10 min. False-positive results were avoided by following the standard precautionary measures for PCR [14].

1. Electrophoresis and gel documentation

The final PCR products/genes were visualised on 1% agarose gel stained with ethidium bromide using ultraviolet light, following electrophoresis at 100 volts for 40 min.

1. Sequence determination

The PCR product was purified using ExoSAP-IT™ (Affymetrix Inc., Santa Clara, California, USA), according to the manufacturer's guidelines. The purified product was sequenced in both forward and reverse direction using the BigDye Terminator v3.1 Cycle Sequencing Kit (Applied Biosystems), according to the manufacturer's guidelines. The labelled product was then cleaned with the Zymo DNA Clean & Concentrator™−5 kit (Zymo Research Corporation, Irvine, California, USA), with subsequent analysis on an ABI3500XL genetic analyser with a 50 cm array (Applied Biosystems Inc.), using POP-7™.

1. Phylogenetic analysis

The forward and reverse complement sequence reads (derived from the reverse sequence file using http://www.bioinformatics.org/sms/rev_comp.html) were combined into one contiguous sequence at the region of overlap of the two, using CAP3 sequence assembly program [15]. The sequence was edited manually using BioEdit Sequence Alignment Editor Version 7.2.6.1 software [16]. The edited sequence was used for a Megablast search (https://blast.ncbi.nlm.nih.gov/Blast.cgi) for highly similar nucleotide sequences in GenBank^®^ database. The N genes of three vaccine strains SRV9 (AF499686), PV (GU992322) and SAD Vnukovo (GU992319) were also included in the list used for phylogenetic analysis of the Monrovia virus. Detailed information about all the sequences included in the phylogenetic analysis is provided in Table 3. The sequences were aligned using the Multiple Sequence Comparison by Log- Expectation (MUSCLE) option provided in the Molecular Evolutionary Genetics Software Version 7 (MEGA7) [17]. Pairwise genetic distances between sequences were estimated using the Kimura two-parameter substitution model [18], (Table 4). The results were used to construct a maximum likelihood tree using MEGA7. The phylogenetic tree was rooted to MOKV genotype 3 (EU293117) and other rabies-related lyssaviruses. Bootstrapping of 1000 replicates was used to statistically evaluate the branching order of the phylogenetic trees. The percentage of replicate trees in which the various strains clustered together is displayed next to the branches. A bootstrap support of 70% was considered significant and sufficient evidence for phylogenetic grouping [19].

**Table 3.** Rabies lyssaviruses included in the phylogenetic analysis of the Monrovia rabies isolates MF765758, MH507336 and MH507337

GenBank Accession No.	Collection (year)	Country	Host species	Lineage
AB094438.1	2002	Kyrgyzstan	*Myotis blythi* (bat)	Outgroup *Lyssavirus*
KC169985.1	2012	France	*Myotis naterreri* (bat)	Outgroup
AY573943.1	2014	Australia	*Pteropus alecto* (bat)	Outgroup
EU293120.1	1981	Republic of South Africa	*Miniopterus* (bat)	Outgroup *Lyssavirus*
KF155004.1	2004	UK	*Myotis daubentonii* (bat)	Outgroup *Lyssavirus*
MF187859.1	1989	France	*Eptesicus serotinus*	Outgroup *Lyssavirus*
KX874609.1	2013	Nigeria	*Canis familiaris* (domestic dog)	Africa 2
KX874612.1	2010	Nigeria	*Canis familiaris* (domestic dog)	Africa 2
KX874613.1	2013	Nigeria	*Canis familiaris* (domestic dog)	Africa 2
MH507336 (this study)	2017	Liberia	*Canis familiaris* (domestic dog)	Africa 2
MH507337 (this study)	2017	Liberia	*Canis familiaris* (domestic dog)	Africa 3
JX193798.1	2009	Tanzania	*Civettictis civetta* (civet cat)	Ikoma lyssavirus (outgroup)
JX197457.1	2012	China	*Murina leucogaster* (bat)	Irkut virus (outgroup)
EF614261.1		Tajikistan	Bat	Khujand lyssavirus (outgroup)
EU293108.1	1985	Senegal	*Eidolon helvum* (bat)	Lagos bat virus (outgroup)
KY006983.1	2011	Spain	*Miniopterus schreibersii*	Lleida bat lyssavirus (outgroup)
GU170201.1	2009	Kenya	*Hipposideros commersoni*	Shimoni bat virus (outgroup)
EF614258.1		Russia	Bat	West Caucasian bat virus (outgroup)
MH481711.1	2018	Liberia	Dog	Africa 2
MH481712.1	2018	Liberia	Dog	Africa 2
MH481713.1	2017	Liberia	Dog	Africa 2
EU853577.1	1986	Tunisia	Human	Africa 1a
EU853580.1	1987	Ethiopia	Bovine	Africa 1a
U22643.1	1982	Algeria	Dog	Africa 1b
U22852.1		Morocco		Africa 1b
KX148209.1	2004	Madagascar	Dog	Africa 1c
KX148210.1	1998	Madagascar	Human	Africa 1c
U22640.1	1990	Niger	Dog	Africa 2
U22639.1	1995	Cote D’ Voire		Africa 2
EU718771.1	2006	Chad	Dog	Africa 2
KX148242.1	1994	Cameroon	Dog	Africa 2
KF022180.1	2005	Nigeria	Dog	Africa 2
EU888728.1	2006	Nigeria	Dog	Africa 2
AF467949	2002	Republic of South Africa	Mongoose	Africa 3
DQ837431.1	1950	Israel	Dog	Africa 4
DQ837461.1	1998	Egypt	Dog	Africa 4
KU963489.1	2005	China	Dog	Asia (China 2)
DQ666322	2008	China	Dog	Asia (China 2)
MF765758 (this study)	2006	Liberia	Dog	Asia
U22482	1986	Iran	Dog	Asia
U22918	1989	Nepal	Dog	Asia
U22480	1990	Oman	Red fox	Asia
U22654	1981	Greenland	Arctic fox	Oceania
U22475	1991	Germany	Red fox	Europe/Mid. East
U42706	1986	Bosnia-Herzegovina	Red fox	Europe/Mid. East
EU293117	1974	Cameroon	*Crocidura sp*. (shrew)	Mokola lyssavirus (outgroup)
GU992322	1993	Morocco		*Rabies virus* strain Pasteur (PV)
GU992319	1995	Czech Republic		Vaccine strain SAD Vnukovo

**Table 4.** Estimates of evolutionary distances between N gene sequences of selected rabies lyssaviruses available in NCBI GenBank and Monrovia rabies lyssavirus isolate MF765758.

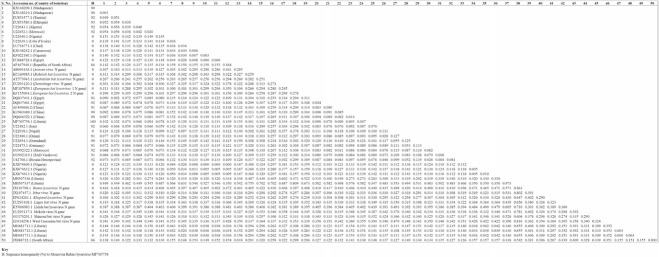

In view of limitations encountered in the use of MEGA for phylogenetic analysis of particularly short sequences, a complementary phylogram of the detected RABV N-sequences was generated using the Maximum Likelihood algorithm and subtree-pruning-regrafting branch-swapping option in PhyML version 3.0 (PhyML3) software [20].

## Results

### Gel documentation imagery

**Fig. 2.**
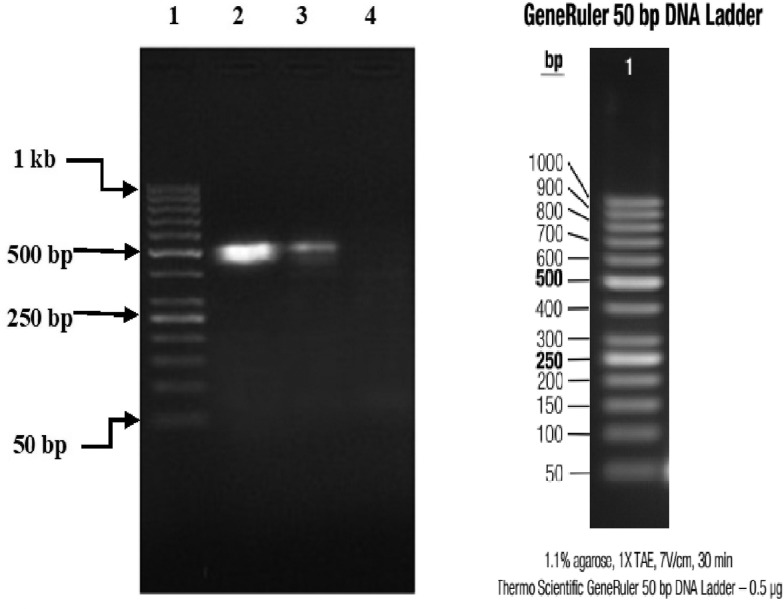
Partial amplification products of Lyssavirus N gene on 1% agarose gel, visualised with UV light; lane 1, marker (50 base pairs DNA ladder); lane 2, Monrovia dog brain sample (Lab Id. No: CCPZUI/DBS/01); lane 3, positive control; lane 4, negative control.

**Fig. 3.**
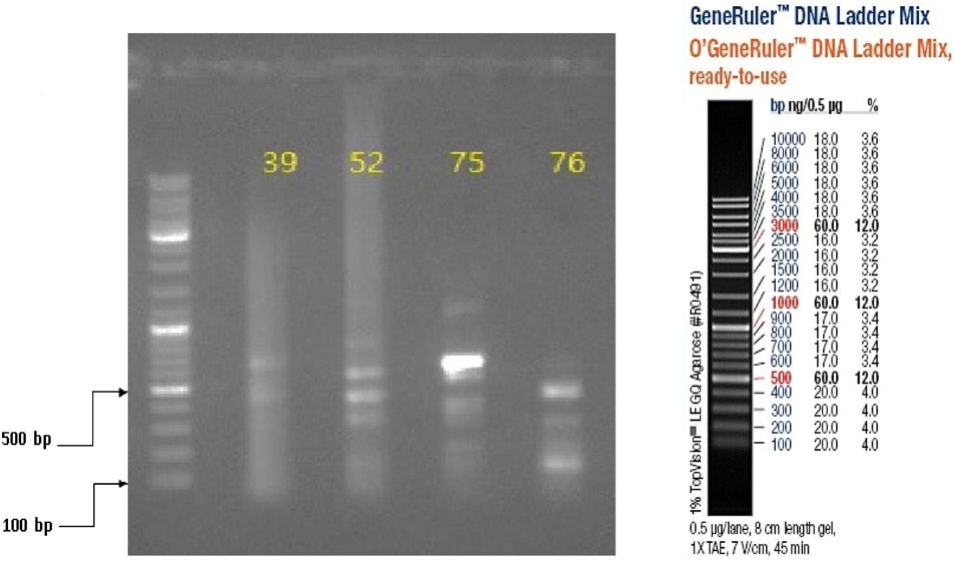
Partial amplification products of Lyssavirus N gene on 1% agarose gel, visualised with UV light; in lane 1 is the 100 bp DNA ladder while in lanes 2–5 are Monrovia samples nos. DBS/039, DBS/052, DBS/075 (GenBank Accession No. MH507336) and DBS/076 (GenBank Accession No. MH507337), respectively.

Three (15%) of 20 specimens (19 brain samples of suspected rabid dogs and one human saliva) tested were positive for RABV N gene by RT-PCR. The gel documentation of PCR products of the three positive genes are shown in Figures 2 and 3. Each gene was within 550–650 bp size range. The DBS 01, DBS 075 and DBS 076 sequences were 554, 171 and 126 bp ORF, respectively.

### Partial N gene characteristics

**Fig. 4.**
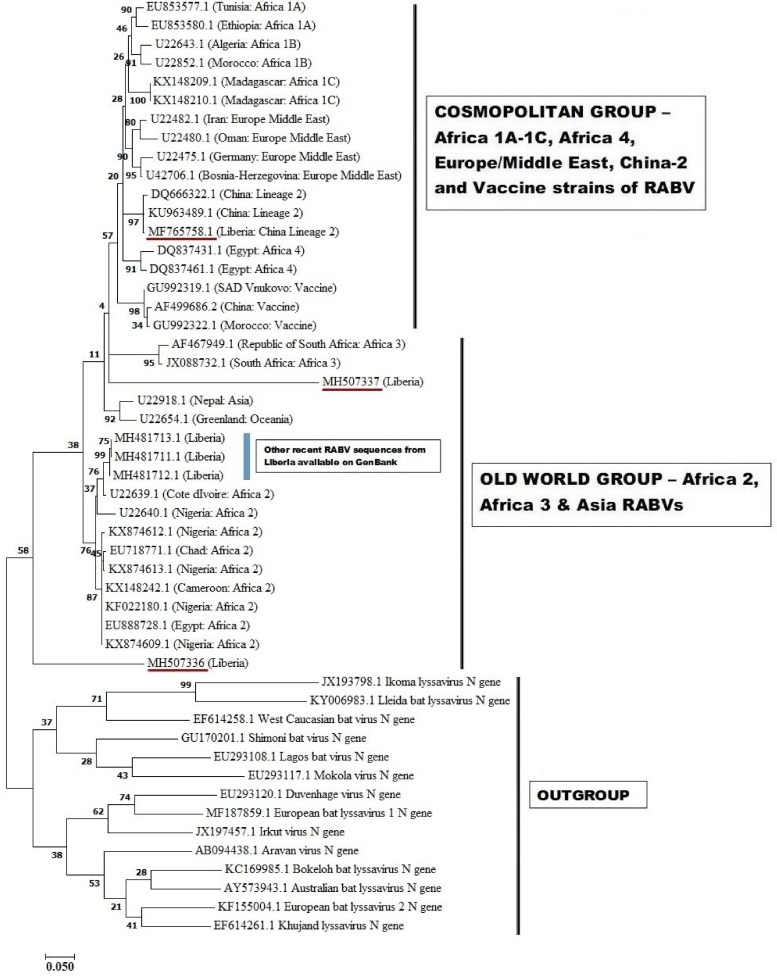
Maximum likelihood (ML) phylogenetic tree of the first Monrovia RABV N-gene isolate (GenBank Accession No. MF756758) generated using ML algorithm (1000 bootstrap replications). The analysis involved 50 nucleotide sequences. All positions with <95% site coverage were eliminated. Evolutionary analyses were conducted in MEGA7 [17]. The bootstrap values (%) are shown next to the branches. The tree is drawn to scale, with branch lengths in the same units as those of the evolutionary distances used to infer the phylogenetic tree. The evolutionary distances were computed using the Kimura two-parameter method [18] and are in the units of the number of base substitutions per site.

These three sequences were deposited in GenBank, under accession numbers MF765758, MH507336 and MH507337, respectively. The Monrovia RABV sequence MH765758 had very close resemblance (99% homology) with China lineage 2 RABV strains KU963489 (or SN2-62-CanineCHINA2005) and DQ666322 (or Jiangsu_Yc63), previously isolated from dogs in China (Table 4). The second RABV sequence obtained in Monrovia MH507336 clustered with Africa lineage 2 RABVs from Côte d'Ivoire, Nigeria, Chad, Cameroon and Egypt, while the third RABV sequence MH507337 clustered with Africa lineage 3 RABVs AF467949 and JX088732 (Fig. 4). Africa lineage 3 RABVs were previously reported only in Southern Africa viverrids [21].

**Fig. 5.**
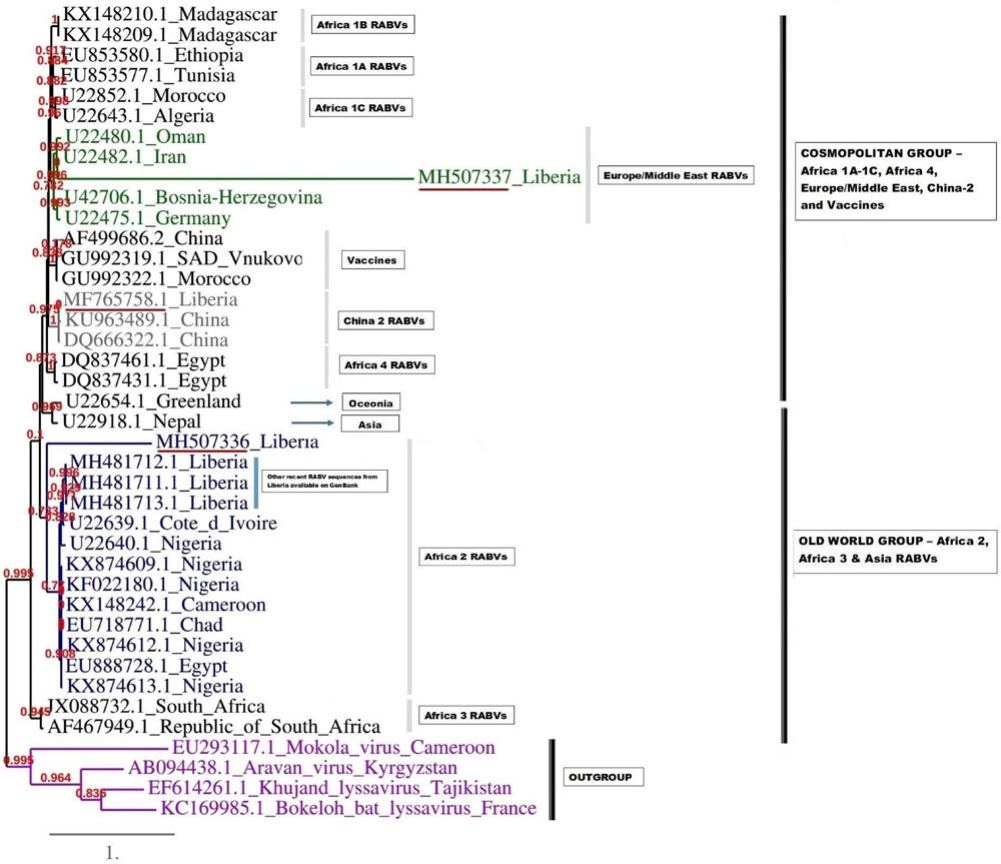
Maximum likelihood (ML) phylogenetic tree of the MH507336 and MH507337 generated using ML algorithm. The analysis involved 39 nucleotide sequences. Evolutionary analyses were conducted in PHYML3 [20]. The bootstrap values (%) are shown next to the branches. The tree is drawn to scale, with branch lengths in the same units as those of the evolutionary distances used to infer the phylogenetic tree. The evolutionary distances were computed using the Kimura two-parameter method [18] and are in the units of the number of base substitutions per site.

A complementary (second) phylogenetic tree generated using PhyML3 is shown in Figure 5. Again, the first Monrovia sequence MF765758 clustered with China strain, KU963489 (canine origin) and DQ666322 (canine origin). The MH507336 sequence segregated along with Africa lineage 2 RABVs strains, while MH507337 clustered with RABVs of Europe/Middle East lineage including U22480.1 (Oman), U22482.1 (Iran) U22475.1 (Germany) and U42706 (Bosnia-Herzegovina) (Fig. 5).

## Discussion

This study was aimed at detecting and characterizing RABV strains circulating in Monrovia city and environs in Liberia, West Africa, 2016–2017, by RT-PCR testing of archived brain specimens of suspected rabid dogs using primers specific for the N gene of lyssaviruses, followed by sequencing analysis of the amplicons obtained. Three strains of RABV sequences were detected and accessioned in NCBI GenBank. All three isolates were from the Red Light area of Paynesville, a commercial district and notable slum located in the suburb of Monrovia, the capital city of Liberia. While the first report of DTHR in Liberia dates back to the 1950s [2], there are no reports on molecular characteristics of RABVs from Liberia that predate our characterisation of the three Monrovia RABV strains that were identified in this study. The co-circulation of China lineage-2 with Africa lineage-2 and lineage-3 was probably due to inadvertent importation of rabies virus into Liberia from China or neighbouring countries, as a result of detection failure along import barriers. This allusion is plausible with recent findings that show the predominance of China sourced rabies vaccines in Nigeria [22] and the all-time high Chinese presence in Liberia; while rabies vaccine quality compromise has been widely reported in China with resultant rabies outbreak following dog bite in humans [23, 24]. The direct importation of dogs considered vaccinated, yet incubating rabies is the most likely source of introduction of the China lineage 2 RABV into Liberia.

The phylogenetic results of this study revealed similarity of the Monrovia RABV MF765758 with DQ666322 or Jiangsu_Yc63 (China lineage 2) and other RABV lineages including Africa 1A-1C, Africa 4, Europe/Middle East and Asia, and vaccine strains PV (GU992322), SRV9 (AF499686) and SAD Vnukovo (GU992319) (Figs 4 and 5). The Chinese lineage C (especially C1) RABVs are closely related to rabies vaccine strains PV and SAD [25]. Incidentally, antirabies vaccines that are used for dog immunisations in Monrovia and environs in Liberia are the inactivated Czech strains SAD Vnukovo-32 and PV Op VB. Clustering of the Liberian RABV strain MH507337 with RABV strain AF46794 regarded as a mongoose adapted strain previously isolated only in the southern African region [21], constitutes another unique finding of this study, and warrants further detailed investigation to ascertain its host species and transmission mechanisms in Monrovia. It is also necessary to determine whether MF765758 the Chinese strain discovered in a Monrovia dog resulted in a self-terminating outbreak or perpetual propagation.

The short strands of MH507336 and MH507337 genes accounted for their sub-optimal positioning on the phylogram. This limitation was further resolved in PhyML3 which presents a robust platform and ML algorithm applicable to the phylogenetic analysis of RABVs [26]. The recent discovery of three newly accessioned rabies N-genes from Liberia obtained from specimens collected through the CVLC in Monrovia, through an ongoing multi-national effort [27], further clarified the phylogenetic relationship of these sequences. These latest RABV sequences segregated along with others that belong to Africa lineage 2 (Figs 4 and 5). This corroborates with predominance of Africa lineage 2 strains that are indigenous to West Africa being in circulation in Liberia.

Phylogenetic studies have shown that RABV isolates tend to form distinct groups and patterns of distribution that are each associated with specific geographical regions of the world [28]. Africa lineage 1 RABVs closely resemble RABVs from Europe and Asia and are thought to be recently introduced into Africa from Europe [29]. On the other hand, Africa lineage 2 RABVs comprise wild-type strains that originated from several central and eastern African countries, and are ancestral to Africa 1 and Eurasian RABVs [30]. The phylogenetic evidence gathered in this study suggests that the Monrovia RABV isolate (MF765758) has its origin in Asia, rather than it being an autochthonous (Liberian) RABV or an extant strain from another African country. Till date, only Africa lineages 1 and 2 RABVs have been isolated from rabid dogs in West and Central Africa [31–33], and to the best of our knowledge, this is the first report from any African country of a RABV isolate of domestic dogs that is phylogenetically closely related to RABVs circulating in China.

It is plausible that other lyssaviruses apart from RABV are involved in recurrent outbreaks of rabies in Monrovia and other parts of Liberia because more than 56 different species of bats have been documented in different ecological zones of the country [34], and all lyssaviruses with the exception of MOKV and IKOV are maintained in bats [35]. In neighbouring West African countries such as Nigeria and Ghana, enzootic LBV exposures have been detected in *Megachiroptera* bats especially *Eidolon helvum* [36, 37]. Although there are no reported spill-overs of LBV into humans, LBV infections have been detected in dogs [38] and wildlife such as mongoose [12]. Such infections are of concern to public health authorities because of the likelihood that lyssavirus infections such as LBV may yet spill-over into the human population in future, and the available vaccines against RABV genotype 1 offer no protection against LBV.

While the goal of achieving a more effective and efficient detection, surveillance and stepwise elimination of animal and human rabies in West African cities and villages deserves stronger collaboration among African scientists, governments and beyond, current trends on epidemiological reports on one-health action against rabies presented at the 5th International Conference on Rabies in West Africa (RIWA), Bamako, Mali, 23–25 October 2018 show gradation in rabies one-health actions within the Economic Community of West African States (ECOWAS countries). Retrospective studies of DBVs presented for rabies exposure treatment in Buchanan, Suakoko and Voinjama, 2008–2013 showed that more than half of the DBVs were below 20 years of age, mostly male [3]. They were comparable to what obtained in Bamako city, Mali. Finally, while it was shown that canine rabies transmission to humans can be interrupted in an African city with currently available dog rabies vaccines, provided that the vaccination area includes larger adjacent regions, and local communities are informed and engaged [26], the higher education participation concept for developing more effective rabies surveillance and systematic elimination through mentorship programme in collaboration with one-health activities of ECOWAS governments and through Regional Rabies Control Strategies Elaboration and Validation Workshop is critically important to achieve the 2030 goal of elimination of rabies in Africa.

## Conclusions

This study is the first to confirm *RABV* infection by molecular diagnostic technique in Liberia. A canine rabies virus variant likely to have been imported from Asia was found co-circulating with Africa lineages 2 and 3 RABVs at the dog–human interface in Monrovia, Liberia, during outbreaks of DTHR that occurred between 2016 and 2017. While there is a need to investigate the yet unknown involvement of wildlife in the maintenance and spread of rabies in Liberia, efforts should be focused on improving rabies surveillance and reporting in Liberia. An in-country rabies diagnostic laboratory that complies with the World Organization for Animal Health procedures should be established in Liberia. The current findings should stimulate more efforts towards rabies active surveillance in one-health mode within the Economic Community of West African States, particularly within the Regional Rabies Control Strategies Elaboration and Validation Workshop.
